# Actin like-6A promotes glioma progression through stabilization of transcriptional regulators YAP/TAZ

**DOI:** 10.1038/s41419-018-0548-3

**Published:** 2018-05-03

**Authors:** Jianxiong Ji, Ran Xu, Xin Zhang, Mingzhi Han, Yangyang Xu, Yuzhen Wei, Kaikai Ding, Shuai Wang, Bin Huang, Anjing Chen, Di Zhang, Zheng Jiang, Shuo Xu, Qing Zhang, Wenjie Li, Shilei Ni, Jian Wang, Xingang Li

**Affiliations:** 1 Key Laboratory of Brain Functional Remodeling, Department of Neurosurgery, Qilu Hospital of Shandong University and Brain Science Research Institute, Shandong University, 107 Wenhua Xi Road, Jinan, Shandong 250012 China; 2 Brain and Mind Centre, and Faculty of Health Sciences, University of Sydney, Camperdown, NSW 2050 Australia; 3 Department of Neurosurgery, Jining No.1 People’s Hospital, Jiankang Road, Jining, 272011 China; 4 Department of Biomedicine, University of Bergen, Jonas Lies vei 91, Bergen, 5009 Norway

## Abstract

Increased Actin-like 6A (ACTL6A) expression has been implicated in the development of diverse cancers and recently associated with the Hippo signaling pathway, which is known to regulate biological properties, including proliferation, tissue regeneration, stem cell biology, as well as tumorigenesis. Here we first show that ACTL6A is upregulated in human gliomas and its expression is associated with glioma patient survival. ACTL6A promotes malignant behaviors of glioma cells in vitro and in orthotopic xenograft model. In co-immunoprecipitation assays, we discover that ACTL6A physically associated with YAP/TAZ and furthermore disrupts the interaction between YAP and β-TrCP E3 ubiquitin ligase, which promotes YAP protein degradation. Moreover, effects of ACTL6A on glioma cells proliferation, migration, and invasion could be mediated by YAP/TAZ. These data indicate that ACTL6A may contribute to cancer progression by stabilizing YAP/TAZ and therefore provide a novel therapeutic target for the treatment of human gliomas.

## Introduction

Malignant glioma is the most common and aggressive type of brain malignancy in adults^1^. Despite great advancement in therapeutic techniques for treating glioma, such as surgery, radiotherapy, and chemotherapy, patients with malignant glioma still only have an average survival of 12–15 months^2,3^. In the past several years, efforts taken to develop new effective therapeutic targets for glioma have focused on identifying the fundamental molecular changes occured in tumors^4,5^. Current goals are to understand how these changes promote malignant progression^6^.

Actin-like 6A (ACTL6A), known as BAF53A, is a subunit of SWI/SNF (BAF) complex. It is involved in various stem cell function, including chromatin remodeling, transcriptional regulation, and nuclear transition^7–9^. Unlike the specific expression of other SWI/SNF subunits in differentiated tissues, ACTL6A is highly expressed in stem cells and progenitor cells^10,11^. Studies have shown that ACTL6A enforces the progenitor state by promoting cell self-renewal and preventing differentiation^8^. Increased ACTL6A expression has been reported in various cancers, including primary rhabdomyosarcomas, hepatocellular carcinoma, and osteosarcoma^12–14^. Although ACTL6A was characterized as an oncogenic driver in many human cancers^15^, the underlying mechanisms remain limited. It has been reported that ACTL6A interacts with oncoprotein c-Myc and has a role in c-Myc-interacting nuclear complexes^16^. ACTL6A was also found to interact with TP63 and regulate transcription of various key genes in head and neck squamous cell carcinoma (HNSCC), including a Hippo signaling pathway regulator WWC1^17^.

The Yes-associated protein (YAP) and transcriptional coactivator with PDZ-binding motif (TAZ) are two key downstream effectors of the Hippo signaling pathway, which regulates cellular proliferation, organ size, tissue regeneration, and stem cell biology, as well as tumorigenesis^18–20^. In response to a variety of stimuli, YAP/TAZ are dephosphorylated and translocate into the nucleus to regulate transcriptional activities^21,22^. Enhanced YAP/TAZ expression and nuclear accumulation have frequently been observed in various human cancers, including hepatocellular carcinoma, breast cancer, colorectal cancer, and glioma^23–26^. Several clinical studies have indicated that YAP/TAZ was highly expressed in aggressive glioma subtypes (classical and mesenchymal) and their expression is positively correlated with poor overall survival of glioma patients^25,27–29^. Several lines of evidence from in vitro and in vivo studies have suggested that YAP/TAZ have a critical role in gliomagenesis^28,30–32^. YAP/TAZ proteins were identified as oncogenes driven by CD44 to promote glioma progression^33^. More recently, a new crosstalk mechanism between Hippo/YAP and Wnt/β-catenin pathway has been found and has a functional role in glioma growth^30^. Although mass studies have suggested that upregulation of YAP/TAZ is a common feature in glioma, the pathological mechanisms are still poorly understood.

In the current study, we examined ACTL6A expression in primary human glioma tissues and cell lines, and found that ACTL6A is overexpressed relative to normal brain tissues and normal human astrocytes (NHAs). These results were the basis for the design of several functional assays to determine whether ACTL6A promotes glioma progression both in vitro and in vivo, and to evaluate YAP/TAZ in glioma cells as a potential mediator of the oncogenic activity of ACTL6A in human cancer.

## Results

### ACTL6A is highly expressed in primary human gliomas and predicts poor prognosis

**Fig. 1:**
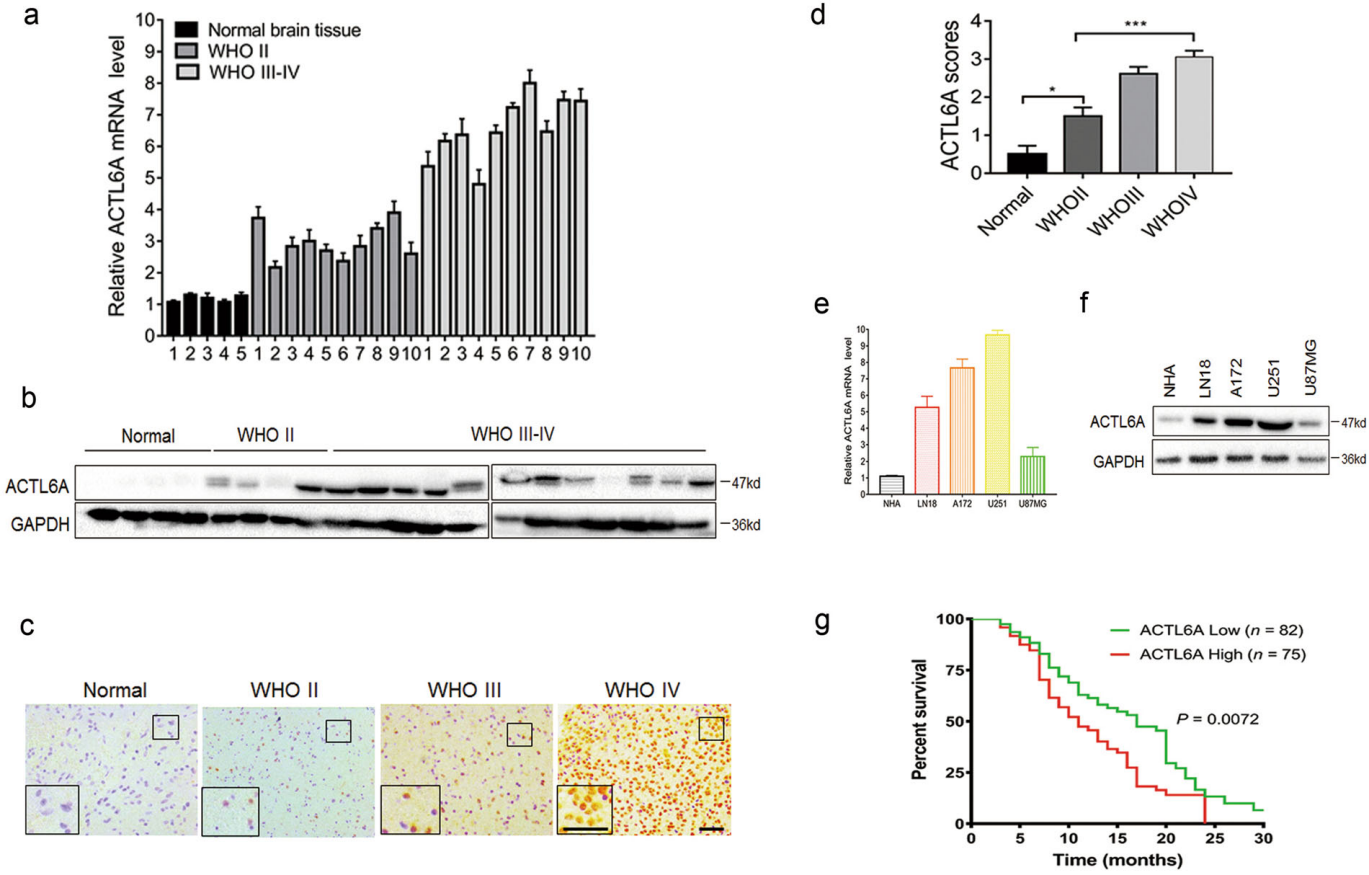
ACTL6A is overexpressed in glioma patient samples and cell lines. **a** qRT-PCR performed on primary human glioma (*n* = 20; WHO grade II–IV) and non-neoplastic (*n* = 5) tissue samples. Data are represented as the mean ± SEM. **b** Western blot analysis of ACTL6A levels in lysates prepared from different grades of human gliomas (WHO grades II–IV) and normal brain tissues. **c** Representative images of IHC staining of ACTL6A in human glioma and non-neoplastic tissue samples. Scale bars, 100 µm. **d** Graphical representation of scoring performed on IHC staining of glioma and non-neoplastic tissue samples for ACTL6A. Data are represented as the mean ± SEM. **e**, **f** qRT-PCR and western blot analysis of ACTL6A levels in NHA, LN18, A172, U251, and U87MG cell lines. GAPDH was used as a loading control. **g** Kaplan–Meier survival analysis performed with survival data of glioma patients with high ACTL6A expression (*n* = 75) vs. low ACTL6A expression (*n* = 82). Log-rank test, *P* = 0.0072. Student’s *t*-test: *P < 0.05, ***P < 0.001

To understand the role of ACTL6A in the development of human glioma, we first examined RNA and protein levels in primary human glioma samples and cell lines. RNA was prepared from primary gliomas (*n* = 20; World Health Organization (WHO) grade II–IV) and non-neoplastic brain tissue samples (*n* = 5), and quantitative reverse transcription-PCR (qRT-PCR) was performed. Compared with normal brain tissues, the mRNA level of ACTL6A was increased in gliomas with highest expression in grade III–IV cases (Fig. 1a). Correspondingly, ACTL6A protein levels examined by western blotting and immunohistochemical staining in a cohort of glioma and non-neoplastic brain tissue samples from our own institution were increased in high-grade gliomas (WHO III–IV; *n* = 12), relative to normal brain tissues (*n* = 4) and low-grade gliomas (WHO II; *n* = 4; Fig. 1b). Interestingly, although non-neoplastic samples were largely negative, increased ACTL6A expression was found in higher tumor grade (Fig. 1c,d). High expression (> 2) was observed in 2 of 12 low-grade gliomas (WHO II; 16.7%) and in 29 of 48 high-grade gliomas (WHO III–IV; 60.4%). High ACTL6A expression significantly correlated with increased tumor grade (Table 1, *P* < 0.01). Glioma cell lines in vitro also showed increased levels of ACTL6A mRNA and protein relative to a normal cell population, NHAs (Fig. 1e,f). The mRNA and protein levels in U87MG were not increased relative to NHA. Kaplan–Meier survival analysis revealed that glioma patients with higher ACTL6A expression had worse outcome (median survival, 17 months vs. 11 months; high ACTL6A expression and those with low; Fig. 1g).

**Table 1:** Association between ACTL6A expression and clinicopathologic factors in glioma

Variables	No. of cases	*ACTL6A* expression		*P*-value
		Low	High	
Age (year)				
< 60	39	20	19	0.5334
≥ 60	21	9	12	
Gender				
Male	37	17	20	0.8871
Female	23	11	12	
Tumor size (cm)				
< 4	31	14	17	0.3988
≥ 4	29	10	19	
Cystic change				
Absent	25	10	15	0.2750
Present	35	19	16	
Edema				
None to mild	41	21	20	0.2991
Moderate to severe	19	7	12	
WHO grade				
II	12	10	2	0.0067
III	13	19	29	
IV	35			

### ACTL6A is required for proliferation, migration, and invasion of glioma cells in vitro and in vivo

**Fig. 2:**
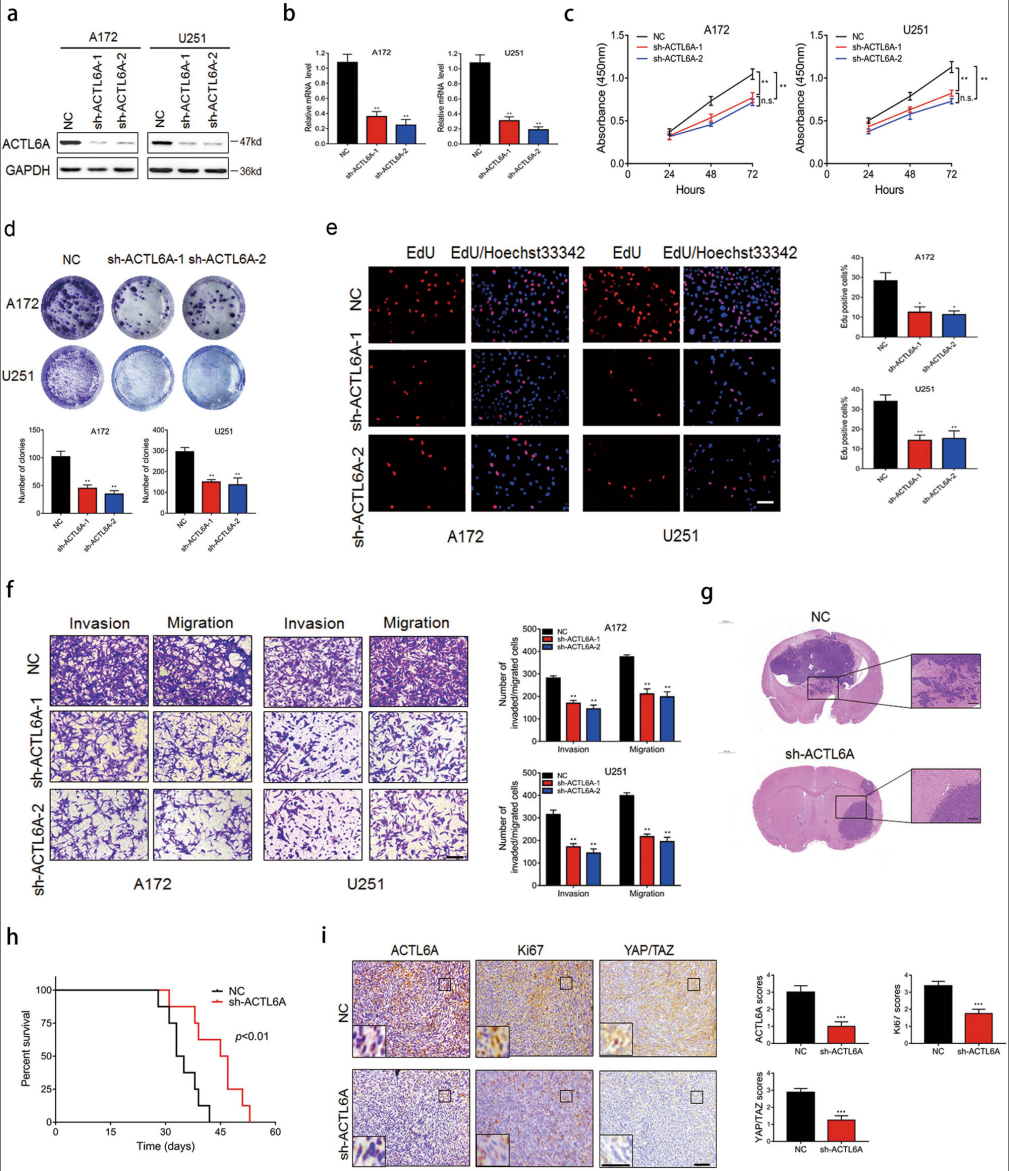
Knockdown of ACTL6A inhibits proliferation, migration and invasion of glioma cells in vitro and in vivo. A172 and U251 cells were infected with ACTL6A shRNAs and knockdown efficiency was determined by both **a** western blotting and **b** qRT-PCR. Data are represented as the mean ± SEM. **c** A172 and U251 cells were infected, and cell viability was analyzed by CCK8 assay. Data are represented as the mean ± SEM. **d** EdU assay for modified A172 and U251 cells. Graphic representation of EdU-positive cells in A172- and U251-NC, sh-ACTL6A-1, and sh-ACTL6A-2 cells. Data are represented as the mean ± SEM. Scale bars, 100 µm. **e** Representative images of colony-forming assay for modified A172 and U251 cells. Cells were fixed and stained, colonies were counted, and results are represented in the bar graph. Data are represented as the mean ± SEM. **f** Representative images of fixed and stained transwell migration and invasion assays performed on modified A172 and U251 cells. Graphic representation of migrated and invaded cells counts from transwell assay. Data are represented as the mean ± SEM from three independent experiments. Scale bars, 100 µm. **g** Representative HE staining of orthotopic xenografts from U251-NC and -sh-ACTL6A groups. Scale bars, 1000 µm, 200 µm. **h** Kaplan–Meier survival analysis performed with survival data of mice implanted with U251-NC and -sh-ACTL6A cells. Log-rank test, *P* < 0.01. **i** Representative images of IHC staining of ACTL6A, Ki67, YAP/TAZ levels in xenograft sections from NC and sh-ACTL6A group. Graphic representation of IHC scoring of ACTL6A, Ki67, YAP/TAZ levels in xenograft sections from indicated group. Data are represented as the mean ± SEM. Scale bars, 100 µm. Student’s *t*-test: n.s. = not significant, *P < 0.05, **P < 0.01, ***P < 0.001

**Fig. 3:**
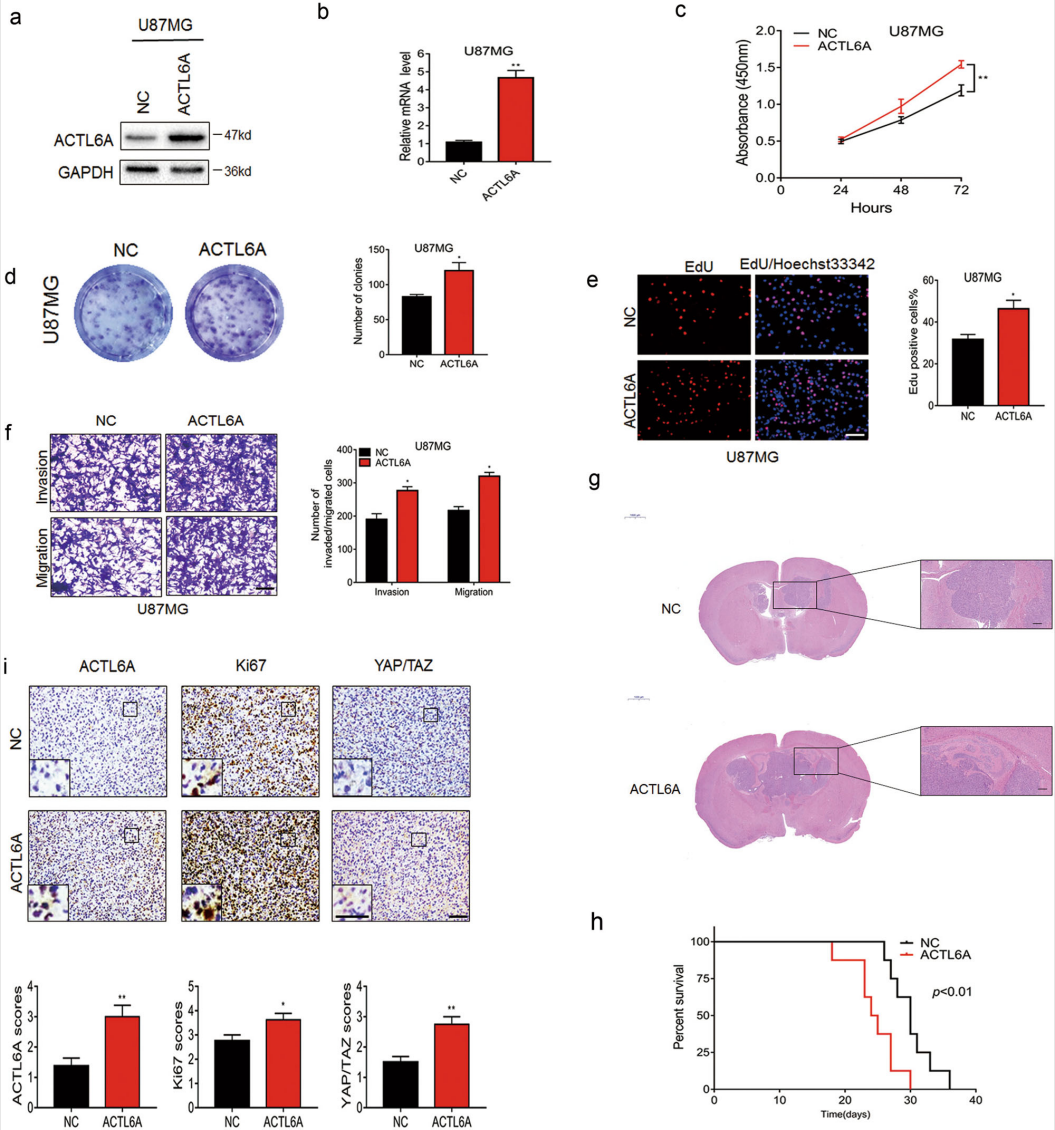
ACTL6A promotes proliferation, migration and invasion of glioma cells in vitro and in vivo. U87MG cells were transfected with empty or ACTL6A-expressing lentivirus and overexpression efficiency was confirmed by both **a** western blotting and **b** qRT-PCR. Data are represented as the mean ± SEM. **c** CCK8 assay performed on U87MG-NC and -ACTL6A. Data are represented as the mean ± SEM. **d** EdU assay for U87MG-NC and -ACTL6A cells. Graphic representation of EdU-positive cells in U87MG-NC and -ACTL6A cells. Data are represented as the mean ± SEM. Scale bars, 100 µm. **e** Colony-forming assay for U87MG-NC and -ACTL6A. Colonies for each condition were quantified and presented in a bar graph. Data are represented as the mean ± SEM. **f** Representative images of transwell migration and invasion assays performed in U87MG -NC and -ACTL6A cells. Graphic representation of migrated and invaded cells counts from transwell assay. Data are represented as the mean ± SEM from three independent experiments. Scale bars, 100 µm. **g** Representative HE staining of orthotopic xenografts from U87MG-NC and - ACTL6A groups. Scale bars, 1000 µm, 200 µm. **h** Kaplan–Meier survival analysis performed with survival data of mice implanted with U87MG-NC and -ACTL6A cells. Log-rank test, *P* < 0.01. **i** Representative images of IHC staining of ACTL6A, Ki67, YAP/TAZ levels in xenograft sections from NC and ACTL6A group. Graphic representation of IHC scoring of ACTL6A, Ki67, YAP/TAZ levels in xenograft sections from indicated group. Data are represented as the mean ± SEM. Student’s *t*-test: *P < 0.05, **P < 0.01

We next performed a series of experiments to assess the role of ACTL6A in various cellular processes of glioma cells. For these experiments, we took advantage of the fact that protein levels of ACTL6A differed in different glioma cell lines relative to NHA (Fig. 1f). A172 and U251, in which ACTL6A was highly expressed, were chosen for knockdown experiments, whereas U87MG, which showed comparable expression level of ACTL6A, was used for ectopic expression experiments. We first examined the efficiency of our sh-RNAs (sh-ACTL6A-1 and sh-ACTL6A-2) and ACTL6A expression constructs. Based on western blotting and qRT-PCR, sh-ACTL6A-2 showed higher efficiency at knocking down ACTL6A in both A172 and U251 compared with sh-ACTL6A-1 (Fig. 2a,b), and the expression construct led to significant increases in ACTL6A protein and RNA levels in U87MG (Fig. 3a,b).

These modified cell lines were first examined for changes in cell proliferation. Growth curves based on the Cell Counting Kit-8 (CCK-8) assay demonstrated that ACTL6A knockdown significantly decreased cell growth in A172 and U251 cells, whereas ACTL6A overexpression enhanced cell growth in U87MG cells (Figs. 2c and 3c). These findings were further confirmed by the EdU and colony-forming assays (Fig. 2d,e and Fig. 3d,e). Interestingly, ACTL6A knockdown also markedly decreased migration and invasion in A172 and U251 cells, whereas U87MG-ACTL6A exhibited enhanced migration and invasion (Figs. 2f and 3f). Taken together, these results indicated that ACTL6A promotes proliferation, migration, and invasion of glioma cells in vitro.

Finally, the modified cell lines were orthotopically implanted in nude mice to evaluate tumor cell growth in vivo. Xenografts from animals implanted with U251-sh-ACTL6A appeared to be more circumscribed and less invasive than tumors that developed from control U251-NC cells (Fig. 2g). In addition, xenografts from animals implanted with U87MG-ACTL6A appeared to be more invasive than tumors that developed from control U87MG-NC cells (Fig. 3g). Overall survival of animals was enhanced with ACTL6A knockdown (median survival, 34 days vs. 46 days; U251-NC and U251-sh-ACTL6A, respectively; Fig. 2h). Overall survival of animals implanted with U87MG-ACTL6A was decreased (median survival, 30 days vs. 24 days; U87MG-NC and U87MG-ACTL6A, respectively; Fig. 3h). Moreover, immunohistochemical (IHC) staining performed on sections from xenografts showed that Ki67 and YAP/TAZ expression levels were correlated with ACTL6A expression levels (Figs. 2i and 3i). Taken together, these results indicated that ACTL6A promoted tumor cell growth in vivo.

### ACTL6A drives YAP/TAZ activation and nuclear localization

**Fig. 4:**
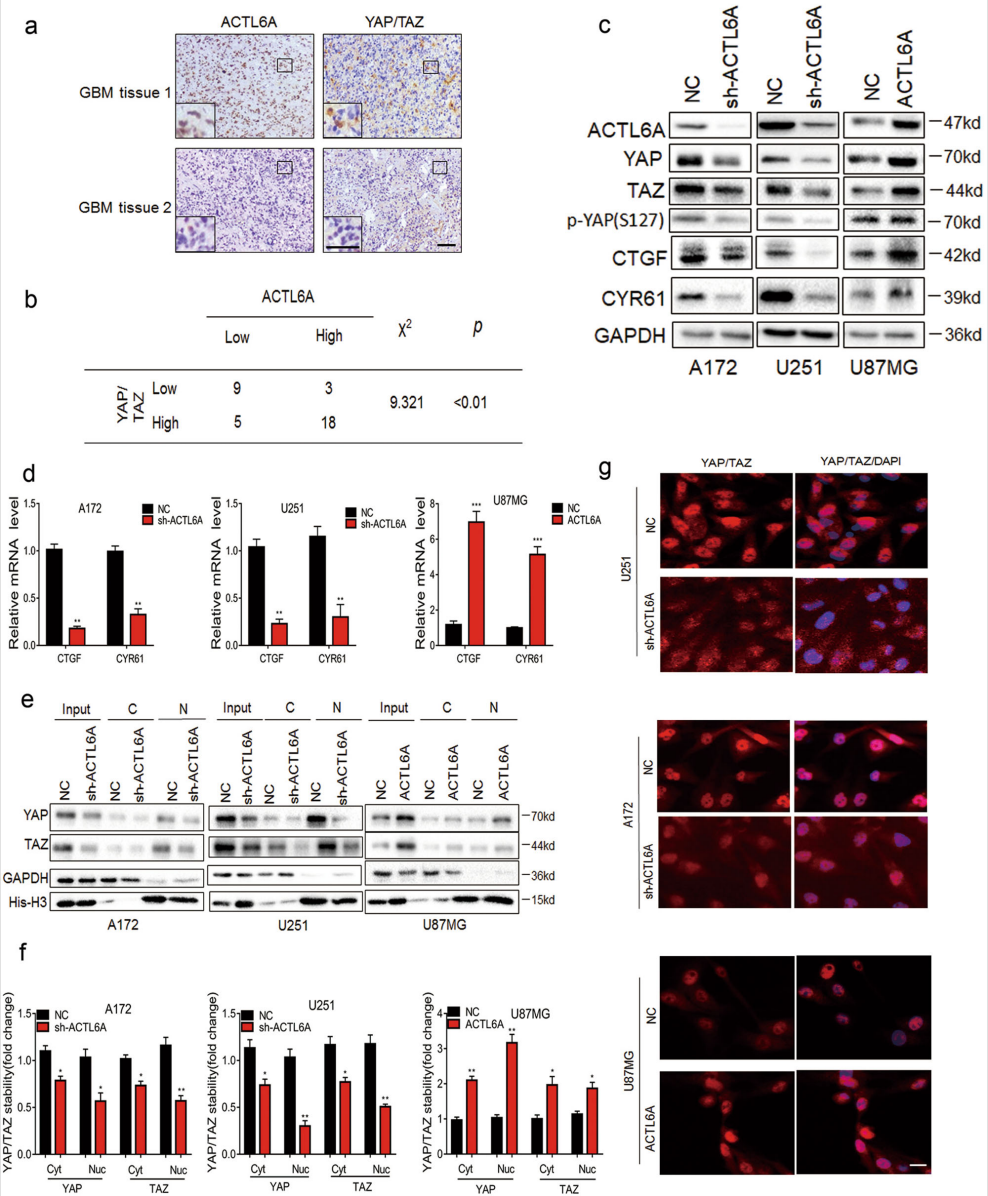
ACTL6A expression increases YA-P/TAZ protein levels and influences their cellular distribution. **a** Representative images of IHC staining of ACTL6A and YAP/TAZ in primary human glioma tissues. Scale bars, 100 µm. **b** Association of ACTL6A expression with YAP/TAZ expression in primary human glioma samples. IHC scores are indicated in parentheses. *χ*^2^-test, *P* < 0.01. **c** Western blot analysis to evaluate YAP/TAZ, their downstream target genes CTGF and CYR61, and YAP phosphorylation in lysates prepared from modified cell lines A172- and U251-NC, -sh-ACTL6A, and U87MG-NC and -ACTL6A. GAPDH was used as loading control. **d** qRT-PCR analysis of CTGF and CYR61 in modified A172, U251, and U87MG cells. Relative expression is shown over GAPDH mRNA. Data are represented as the mean ± SEM. **e** Western blot analysis of cytoplasmic (C) and nuclear (N) fractions prepared from indicated cells. **f** Graph showing the mean YAP and TAZ levels normalized to cytoplasmic (GAPDH) and nuclear (Histone-H3) markers, and then to NC cells. Data are represented as the mean ± SEM. **g** Immunofluorescence of YAP/TAZ in modified A172, U251, and U87MG cells showing cellular localization. Scale bars, 100 µm. Student’s *t*-test: *P < 0.05, **P < 0.01, ***P < 0.001

Based on the functional link established between ACTL6A and Hippo-YAP pathway in squamous cell carcinoma^17^, we investigated whether ACTL6A also regulates activity of YAP/TAZ in human gliomas and thus contributes to the tumor development. In primary glioma samples, IHC scores for ACTL6A correlated with scores for YAP/TAZ, indicating a possible relationship between the proteins (*P* < 0.01; Fig. 4a,b). Western blot analysis of lysates prepared from ACTL6A-modified cell lines revealed that the protein levels of YAP/TAZ and their target genes, *CTGF* and *CYR61*, were correlated with ACTL6A protein levels. YAP/TAZ and downstream genes were reduced in A172- and U251-sh-ACTL6A cells, and increased in U87MG-ACTL6A cells (Fig. 4c). As YAP/TAZ transcriptionally regulates CTGF and CYR61, we also examined RNA levels of these genes in the modified cell lines. Based on qRT-PCR, mRNA levels of CTGF and CYR61 correlated with knockdown or overexpression of ACTL6A in these glioma cell lines (Fig. 4d). To further investigate the relationship between ACTL6A and YAP/TAZ, we examined the effect of ACTL6A on 8 × GTIIC-Lux, a TEAD-dependent YAP/TAZ-responsive reporter. We found ACTL6A depletion strongly decreased the activity of 8 × GTIIC-Lux, whereas ectopic expression of ACTL6A markedly induced the activity, indicating ACTL6A regulates YAP/TAZ activity (Supplementary Figure S1a).

Phosphorylation of YAP at Ser-127 is essential for its association with 14-3-3 and its cytoplasmic retention^19^. Interestingly, we found that phosphorylation of YAP on Ser-127 was reduced in A172- and U251- sh-ACTL6A cells, but relatively unchanged from controls in U87MG-ACTL6A cells (Fig. 4c). However, the ratio of p-YAP(S127) to total YAP was increased in A172- and U251- sh-ACTL6A cells, while decreased in U87MG-ACTL6A cells (Supplementary Figure S1b). To test whether ACTL6A regulates YAP/TAZ transcriptional activity by promoting nuclear localization, protein levels of YAP/TAZ in cytoplasmic and nuclear cellular fractions were evaluated, respectively. (Fig. 4e,f). In A172- and U251-sh-ACTL6A cells, YAP/TAZ levels were decreased in both the cytoplasm and nucleus compared with controls, but were significantly upregulated in both cellular locations in U87MG-ACTL6A. Notably, we found that ectopic expression of ACTL6A promoted nuclear localization of YAP/TAZ, while depletion of ACTL6A showed the opposite effect (Supplementary Figure S1c). The results were further confirmed by immunofluorescence (Fig. 4g). These data indicated that ACTL6A regulates the activity and cellular distribution of YAP/TAZ in glioma cell lines in vitro.

### ACTL6A stabilizes YAP by disrupting YAP-β-TrCP interaction

**Fig. 5:**
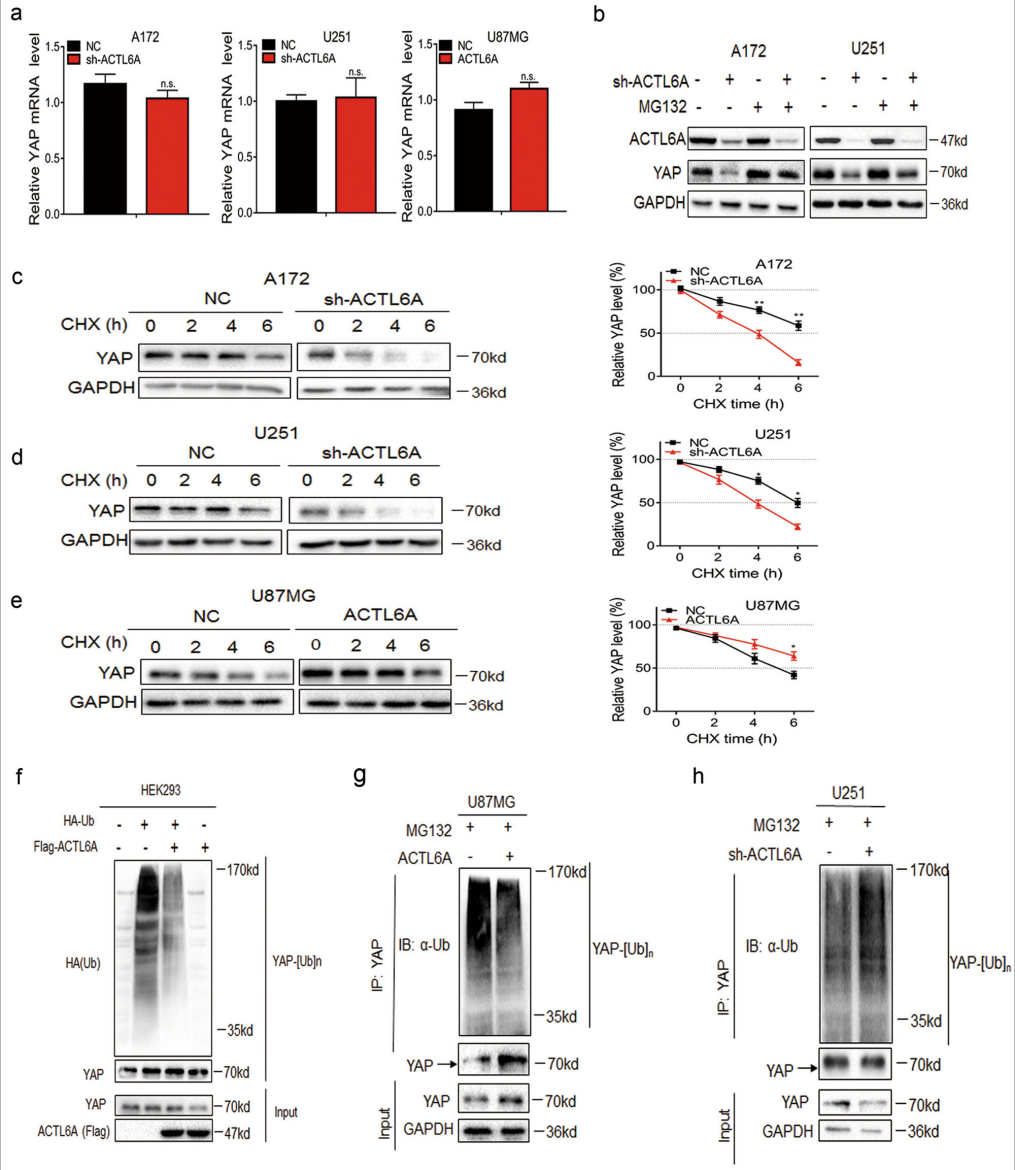
ACTL6A stabilizes YAP proteins. **a** qRT-PCR analysis of YAP in A172- and U251-NC, -sh-ACTL6A, and U87MG-NC and ACTL6A cells. Expression is normalized to GAPDH mRNA. Data are represented as the mean ± SEM. **b** Western blot analysis to evaluate YAP levels in A172- and U251-NC, and -sh-ACTL6A cells after MG132 (20 µM) treatment for 8 h. GAPDH was used as loading control. **c**, **d**, **e** Western blot analysis of YAP protein in modified A172, U251 and U87MG cells treated with CHX (25 µg/mL) for the indicated time. Line graph shows YAP levels normalized to GAPDH at the indicated time points (*n* = 4). Data are represented as the mean ± SEM. **f** Western blot analysis of YAP IPs performed on lysates prepared from HEK293 cells transfected with HA-ubiquitin and plus or minus Flag-ACTL6A and treated with MG132 (20 µM) for 8 h. **g**, **h** Western blot analysis of YAP IPs performed on lysates prepared from U251-NC, -sh-ACTL6A, and U87MG-NC and -ACTL6A cells treated with MG132 (20 µM) for 8 h to examine endogenous YAP ubiquitination. Student’s *t*-test: n.s. = not significant, *P < 0.05, **P < 0.01

To explore the mechanisms by which ACTL6A regulates YAP/TAZ activity, we first examined whether ACTL6A regulates YAP/TAZ transcriptionally. However, YAP/TAZ mRNA levels was not significantly different in A172- and U251-shACTL6A or U87MG-ACTL6A cells compared with control cells (Fig. 5a and Supplementary Figure S2a). Therefore, we next tested whether ACTL6A modulates the stability of YAP/TAZ protein. To test this possibility, we examined YAP/TAZ protein levels in modified cell lines treated with the proteasome inhibitor MG132. We found that MG132 partially reversed the downregulation of YAP/TAZ protein in A172- and U251-shACTL6A (Fig. 5b and Supplementary Figure S2b). In addition, the protein synthesis inhibitor cycloheximide (CHX) was used. The half-life of YAP/TAZ was decreased from ~ 6 h in control cells to ~ 4 h in A172- and U251-shACTL6A cells (Fig. 5c,d and Supplementary Figures S2c and d). Under ectopic expression of ACTL6A, the half-life of YAP/TAZ was extended from ~ 5 h in control cells to > 6 h in U87MG-ACTL6A (Fig. 5e and Supplementary Figure S2e). Finally, endogenous YAP/TAZ ubiquitination was increased in U251-sh-ACTL6A cells, whereas decreased in U87MG-ACTL6A and HEK293 cells when co-transfected with ACTL6A (Fig. 5f,g,h and Supplementary Figures S2 f and g). To rule out potential nonspecific immunoprecipitated smears, we used short hairpin RNAs (shRNAs) to knock down YAP/TAZ in U87MG cells and polyubiquitination levels of YAP/TAZ were evaluated (Supplementary Figure S6). These results indicated that ACTL6A could prevent YAP/TAZ proteins from proteasomal degradation.

**Fig. 6:**
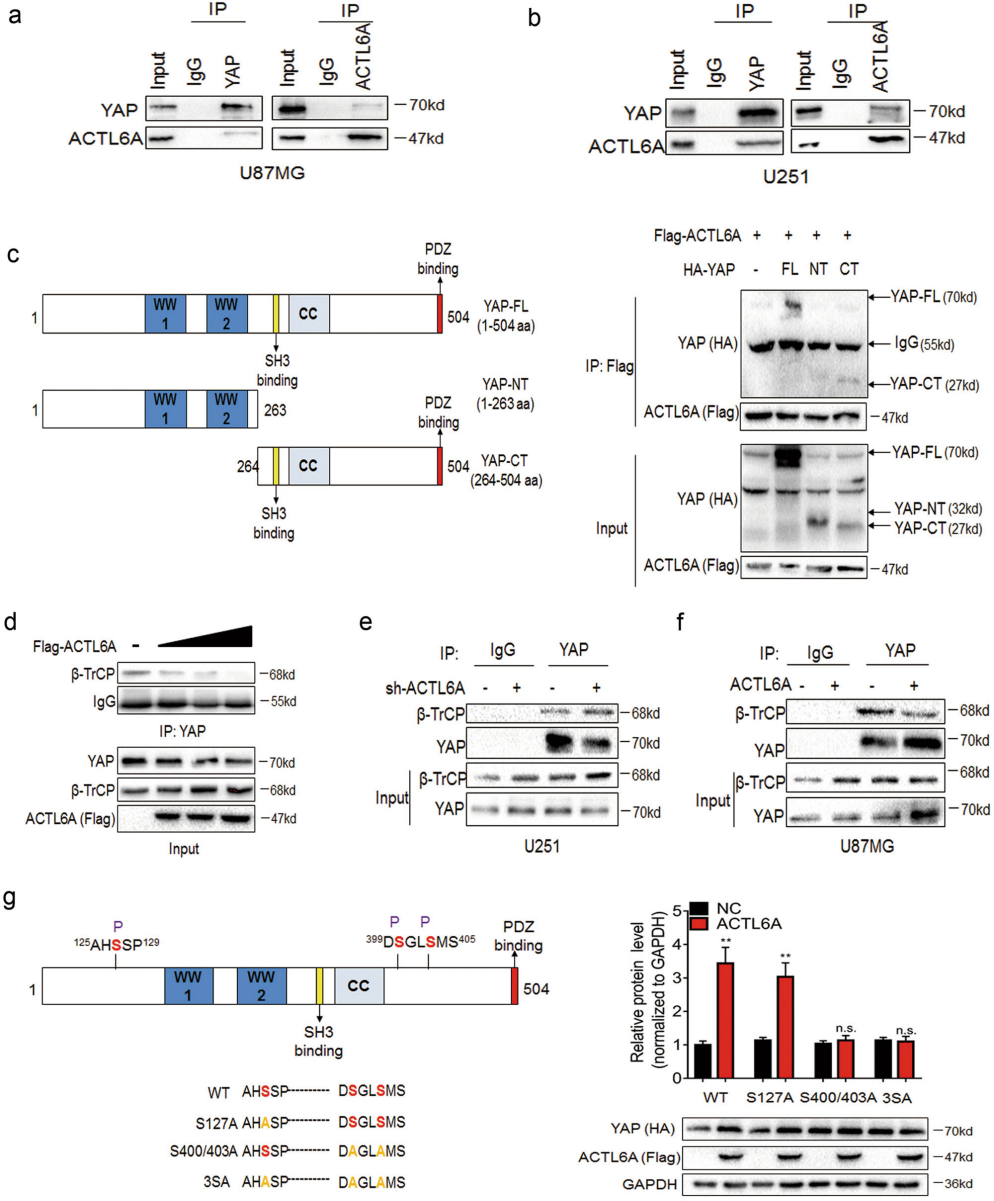
ACTL6A physically interacts with YAP and inhibits protein degradation partially by β-TrCP. **a**, **b** Western blot analysis of co-precipitating proteins in IPs performed using anti-YAP or -ACTL6A antibody on lysates prepared from U251 and U87MG cells. **c** The design of YAP depletion constructs is based on known functional domain. Interactions were analyzed by IP assay. **d** Western blot analysis of YAP IPs performed with lysates prepared from cells transfected with increasing amounts of Flag-ACTL6A. **e**, **f** Western blot analysis of YAP IPs performed with U251-NC and -sh-ACTL6A or U87MG-NC and -ACTL6A cells treated with MG132 (20 µM) for 8 h. **g** The design of YAP mutant constructs. Western blot analysis of YAP mutant protein levels plus or minus Flag-ACTL6A. Student’s *t*-test: n.s. = not significant, **P < 0.01

To determine the mechanism by which ACTL6A promotes the stability of YAP/TAZ, we performed a series of immunoprecipitations (IPs) bringing down complexes with antibodies against YAP/TAZ or ACTL6A. Both YAP/TAZ and ACTL6A were found in complexes with antibodies against YAP or ACTL6A (Fig. 6a,b and Supplementary Figure S3a), indicating that YAP and TAZ are co-regulated by ACTL6A. We therefore chose to focus on regulation of YAP expression in subsequent experiments. We found that C-terminal region of YAP was required for interaction with ACTL6A (Fig. 6c). Our goal was to demonstrate whether ACTL6A associates with YAP and prevents it from binding to β-TrCP, the protein that promotes YAP degradation^34^. β-TrCP recognizes only one site on the C-terminal region (serine 400/403) of YAP when they are phosphorylated by casein kinase 1/LATS (CK1/LATS)^34^. Meanwhile, β-TrCP recognizes two different sites on the N-terminal (serine 58) or C-terminal (serine 314) region of TAZ when they are phosphorylated by glycogen synthase kinase 3 (GSK3) or CK1/LATS, respectively^35,36^. IPs were therefore performed to examine the interaction between ACTL6A, YAP, and β-TrCP. Indeed, YAP and β-TrCP were found in complexes precipitated down with antibody against YAP. However, β-TrCP in complex with YAP was decreased in lysates expressing increasing levels of FLAG-ACTL6A (Fig. 6d). These results indicated that ACTL6A disrupted the interaction between YAP and β-TrCP. To confirm this finding, IPs were performed with lysates prepared from U251-shACTL6A and U87MG-ACTL6A cells. Again, the results showed that the levels of ACTL6A in cells regulated the amount of β-TrCP in complex with YAP (Fig. 6e,f and Supplementary Figure S3b). However, phosphor-mutant YAP-S127A could still be stabilized by ACTL6A. In contrast, phosphor-mutants S400/403A and 3SA could not be further stabilized by ACTL6A (Fig. 6g). Taken together, these data indicated that ACTL6A stabilizes YAP by inhibiting its degradation by β-TrCP.

### ACTL6A promotes glioma cells proliferation, migration, and invasion via YAP/TAZ

**Fig. 7:**
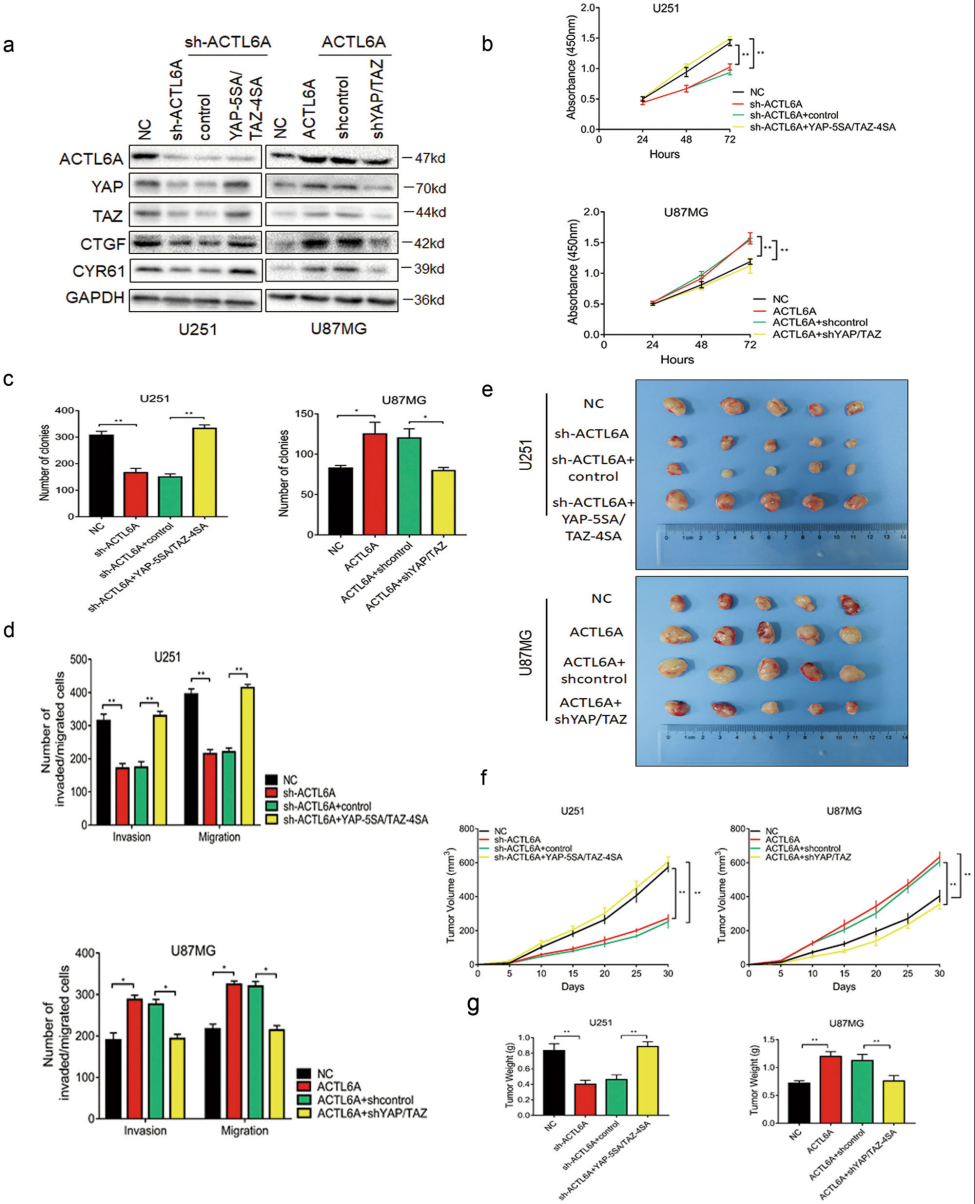
YAP/TAZ mediates ACTL6A-regulated proliferation, migration and invasion in glioma cells. **a** Western blot analysis of lysates prepared from ACTL6A-interfered glioma cells with YAP/TAZ knockdown or active forms ectopic expression. GAPDH was used as loading control. **b** CCK8 assay performed on ACTL6A-interfered glioma cells with YAP/TAZ knockdown or active forms ectopic expression. Data are represented as the mean ± SEM. **c** Colony forming assay performed on ACTL6A-interfered glioma cells with YAP/TAZ knockdown or active forms ectopic expression. Graphic representation of the colony numbers under each condition. Data are represented as the mean ± SEM. **d** Graphic representation of migrated and invaded cells counts from transwell assays performed in ACTL6A-interfered glioma cells with YAP/TAZ knockdown or active forms ectopic expression. Data are represented as the mean ± SEM from three independent experiments. Scale bars, 100 µm. **e** Representative images of subcutaneous xenografts from ACTL6A-interfered glioma cells with YAP/TAZ knockdown or active forms ectopic expression after surgical remove. **f** Tumor growth curves from indicated groups. **g** Tumor weight in nude mice from indicated groups. Student’s *t*-test: *P < 0.05, **P < 0.01, ***P < 0.001

To determine the functional role of YAP/TAZ proteins in the ACTL6A pathway in glioma, shRNAs targeting YAP/TAZ were co-transfected into U87MG-ACTL6A cells, and active forms of YAP (YAP-5SA) and TAZ (TAZ-4SA) were co-transfected into U251-sh-ACTL6A cells, which are constitutively active due to mutations in the inhibitory Lats phosphorylation sites^34,36^. Ectopic expression and knockdown efficiency was evaluated with western blot analysis along with YAP/TAZ target genes, *CTGF* and *CYR61*. Knockdown of YAP/TAZ decreased CTGF and CYR61 expression, and had no significant effect on ACTL6A expression, whereas ectopic expression of YAP-5SA/TAZ-4SA in U251-sh-ACTL6A cells restored the poor CTGF and CYR61 expression due to ACTL6A knockdown (Fig. 7a and Supplementary Figure S4a). Besides, the effects of ACTL6A knockdown on U251 cells could also be rescued by knockdown of β-TrCP (Supplementary Figure S7). Furthermore, ACTL6A-enhanced YAP/TAZ reporter activity was abrogated by YAP/TAZ knockdown, whereas YAP-5SA/TAZ-4SA rescued the effect of ACTL6A depletion (Supplementary Figure S4b). CCK8, colony-forming assays, and transwell assays indicated that the effects of ACTL6A on enhancing proliferation, migration, and invasion were partially reversed by YAP/TAZ knockdown in U87MG cells. In contrast, ectopic expression of YAP-5SA/TAZ-4SA rescued the effect of ACTL6A depletion in U251 cells (Fig. 7b–d and Supplementary Figures S5a, b, c and d). We next investigated the role of ACTL6A in glioma development in the subcutaneous model. The effects of ACTL6A on promoting glioma growth, evaluated by tumor volume and tumor weight, were confirmed and was reversed by YAP/TAZ knockdown. In contrast, ectopic expression of YAP-5SA/TAZ-4SA in U251-sh-ACTL6A cells had the inverse effects (Fig. 7e–g). Collectively, these data indicated that YAP/TAZ may function as a factor downstream of ACTL6A in the development of human glioma.

## Discussion

Here we report that ACTL6A functions as a regulator of YAP/TAZ to promote proliferation, migration, and invasion of human glioma cells. Our data show that ACTL6A is highly expressed in high-grade gliomas relative to low-grade gliomas and non-neoplastic brain tissues. Through knockdown and ectopic expression studies, we found that ACTL6A promotes glioma growth and these effects are reversed by YAP/TAZ, known transcriptional regulators. Mechanistic studies indicated that ACTL6A drives YAP/TAZ nuclear translocation and enhances its transcriptional activity by physically interacting with YAP/TAZ and inhibiting its proteasomal degradation. Finally, we demonstrated that in glioma cells, ACTL6A regulates YAP/TAZ protein stability and downstream events, such as CTGF and CYR61 by disrupting the interaction between YAP and β-TrCP. Therefore, the increase of ACTL6A promotes malignant behavior through stabilization of YAP/TAZ protein and promoting growth genes in the development of human glioma.

Previous studies have already revealed the oncogenic role of ACTL6A in various cancers^12–17^. There are also studies linking ACTL6A to maintenance of a stem cell-like state^7–9^. These findings indicated a functional role of ACTL6A in cancer stem cells. Consistent with these studies, we demonstrated that ACTL6A overexpression strongly promotes glioma cells proliferation, migration, and invasion, which are inhibited by ACTL6A knockdown. These results indicate that ACTL6A may function as an oncogene in human gliomas.

It has been reported that proteins YAP/TAZ are upregulated in diverse cancers^23–26,37,38^. Increased YAP/TAZ expression and nuclear accumulation has been previously reported in human gliomas^25,29,30,33^. Recent work revealed that ACTL6A expression and YAP activation are highly correlated in primary HNSCC and predict poor patient survival^17,39^. In addition, ACTL6A and p63 collaborate as oncogenic drivers in HNSCC through activation of the Hippo-YAP pathway via WWC1^17^. In this study, we confirmed that ACTL6A was significantly associated with YAP/TAZ protein expression in human glioma tissues. In studies performed in vitro, we found that ACTL6A regulates CTGF and CYR61 abundance in glioma cells, accompanied by a nuclear accumulation of YAP/TAZ. In contrast, depletion of ACTL6A significantly decreased YAP/TAZ levels and expression of their target genes. The fact that YAP/TAZ mRNA levels did not change significantly in response to modulation of the expression of ACTL6A, indicated that ACTL6A might regulate YAP/TAZ function through protein degradation in glioma cells.

Stabilization of YAP protein is one of the mechanisms known to control its various functions, including transcriptional activation. Previous studies have demonstrated that recruitment of the β-TrCP ubiquitin ligase facilitates YAP/TAZ ubiquitination and degradation^34^. Here we demonstrated that ACTL6A prevents YAP/TAZ from ubiquitination and prolongs the protein’s half-life. Furthermore, we confirmed that ACTL6A directly associates with YAP/TAZ. We further found that ACTL6A mainly binds to C-terminal region of YAP and inhibits YAP from binding to β-TrCP, which promotes its degradation. However, β-TrCP recognizes two different sites on the N-terminal (serine 58) or C-terminal (serine 314) region of TAZ when they are phosphorylated by GSK3 or CK1/LATS^35,36^; further investigation is required to confirm which terminal region of TAZ ACTL6A mainly binds to. Our results suggest that small molecules designed to prevent ACTL6A from binding to YAP could be promising to interfere the ACTL6A/YAP growth-promoting axis in human tumors.

Based on our results, we suspect that ACTL6A-stablized YAP/TAZ might be responsible for enhanced malignant behaviors of glioma cells. However, we also demonstrated that ACTL6A-mediated glioma cell proliferation, migration, and invasion could be partially reversed by YAP/TAZ knockdown or ectopic expression, indicating that YAP/TAZ is not a unique downstream effector of ACTL6A in glioma cells. ACTL6A has been reported to regulate the activity of many oncogenes, including WWC, SOX2, c-Myc, which all have important roles in promoting tumor progression^13,16,17^. Therefore, it is possible that ACTL6A overexpression in human glioma cells mediates a variety of malignant behaviors through these various oncogenes and pathways.

In conclusion, we found that increased expression of ACTL6A is associated with increasing grade in primary human gliomas. Functional studies demonstrated that ACTL6A overexpression promotes cellular proliferation, migration, and invasion in vitro and in vivo, possibly through the direct interaction and stabilization of transcriptional regulator YAP/TAZ. Our study therefore provides a new insight into the mechanisms governing YAP/TAZ degradation and nuclear accumulation, and possibly an alternative therapeutic target for glioma treatment.

## Materials and methods

### Ethics statement

All experiments and the use of primary human tissue samples were approved by the Research Ethics Committee of Shandong University and performed according to relevant guidelines and regulations. Written informed consent was obtained from all participants. All animal procedures were approved by and performed under the guidance of the Institutional Animal Care and Use Committee of Shandong University.

### Cell culture and transfection

HEK293 cells and human glioma cell lines, U87MG, U251, and A172 were obtained from the Culture Collection of the Chinese Academy of Sciences (Shanghai, China). NHAs were kind gifted by Professor Rolf Bjerkvig, University of Bergen (Norway). All cells were cultured in Dulbecco’s modified Eagle’s medium (DMEM; Life Technologies, USA) supplemented with 10% fetal bovine serum (FBS; Life Technologies). Cells were treated with proteasomal inhibitor MG132 (ApexBio, Hsinchu, Taiwan, China) at 20 µM for 8 h to inhibit proteasomal-mediated degradation and CHX (ApexBio) at 50 µg/mL to inhibit translation.

Transient transfections for plasmids were performed using Lipofectamine 2000 (Invitrogen/Thermo Fisher Scientific). The plasmids used were: pcDNA3.1-ACTL6A-3xFlag, pcDNA3.1-HA-UBB, pcDNA3.1-HA-YAP-wt, pcDNA3.1-HA-YAP-NT, pcDNA3.1-HA-YAP-CT, pcDNA3.1-HA-YAP-S127A, pcDNA3.1-HA-YAP-S400/403A, pcDNA3.1-HA-YAP-3SA, pcDNA3.1-HA-YAP-5SA (S61A, S109A, S127A, S164A, S397A), and pcDNA3.1-HA-TAZ-4SA (S66A, S89A, S117A, S311A) (OBiO Technology, Shanghai, China), pGL3-Control Vector (Promega, Madison, WI, USA), pGL4-SV40 Driven *Renilla* Luciferase Vector (Promega), and 8xGTIIC-luciferase (Addgene, Cambridge, MA, USA). For establishment of stable ACTL6A-knockdown cells, U251 and A172 cell lines were infected with lentivirus containing two different shRNAs targeting ACTL6A (GeneChem, Shanghai, China). U87MG cells were transfected with lentivirus for ectopic expression of full-length *ACTL6A* (GeneChem). U251 cells were transfected with lentivirus for ectopic expression of active forms of *YAP* (YAP-5SA) and *TAZ* (TAZ-4SA) (OBiO Technology). After infection for 48 h, cells were cultured in DMEM containing puromycin (2 µg/mL; Thermo Fisher Scientific) for an additional 2 weeks. The sequences of shRNAs and siRNAs were listed as follow: sh-negative control 5′-TTCTCCGAACGTGTCACGT-3′; sh-ACTL6A-1, 5′-TCCAAGTATGCGGTTGAAA-3′; sh-ACTL6A-2, 5′-GTACTTCAAGTGTCAGATT-3′; shControl, 5′-TTCTCCGAAGGTGTCACGG-3′; shYAP, 5′-GACTCAGGATGGAGAAATTTA-3′; shTAZ, 5′-GCTCATGAGTATGCCCAAT-3′; si- negative control, 5′-UUCUCCGAACGUGUCACGUTT-3′; si-ACTL6A, 5′-GGGATAGTTTCCAAGCTAT-3′; si-YAP, 5′-GACTCAGGATGGAGAAATTTA-3′; si-TAZ, 5′-GCTCATGAGTATGCCCAAT-3′; and si-β-TrCP, 5′-GUGGAAUUUGUGGAACAUC-3′.

### Nuclear fractionation

To determine the subcellular distribution of YAP/TAZ, nuclear and cytoplasmic fractions were isolated using Nuclear and Cytoplasmic Extraction Reagents (Thermo Fisher Scientific), according to the manufacturer’s instructions. Levels of GAPDH and histone H3 were used as loading controls for cytoplasmic and nuclear fractions, respectively.

### Immunohistochemistry

With approval of Ethics Committee of the Qilu Hospital, glioma samples were obtained from 60 patients (WHO II–IV) who had undergone operations at the Department of Neurosurgery. Non-brain tumor specimens (*n* = 6) were collected from brain trauma patients who underwent partial resection of normal brain as decompression treatment for severe head injuries. For staining of ACTL6A, YAP/TAZ, Ki-67, glioma, and normal tissues were fixed with 4% formalin, prepared as paraffin-embedded section. Immunohistochemistry was performed as previous reported^40^. The percentage of positive cancer cells was graded according to the following criteria: 0, no staining; 1, weak staining in < 50% cells; 2, weak staining in ≥ 50% cells; 3, strong staining in < 50% cells; and 4, strong staining in ≥ 50% cells.

### Immunofluorescence and immunoblotting

Immunofluorescence and immunoblotting were performed as previously decribed^40^. The following antibodies were used: ACTL6A, YAP, CTGF, Cyr61, p-YAP, HA, Ki67, Histone-H3 (Abcam, Cambridge, MA, USA), YAP/TAZ, YAP, TAZ, β-TrCP (Cell Signaling Techonology, Beverly, MA, USA), Flag (Sigma, St Louis, MO, USA), Ub, and GAPDH (Santa Cruz Biotechnology, lnc., Dallas, TX, USA).

### Co-immunoprecipitation

Cells were lysed in IP buffer (Pierce, Rockford, USA) containing protease inhibitor cocktail (Sigma). Total protein was incubated with 1–5 µg primary antibodies or IgG and Protein A/G agarose beads (Pierce) overnight at 4 °C with gentle shaking. The immunoprecipitated complexes were then washed with lysis buffer three times and eluted from the beads with protein loading buffer. Western blotting was then performed.

### Cell migration and invasion assay

Cell migration and invasion assays were carried out in uncoated and Matrigel-coated (BD Biosciences, Bedford, MA, USA) Transwell chambers (pore size: 8 μm; Corning Costar, NY, USA). Cells (2 × 10^4^) were added to the upper chamber and assay medium (600 µL medium containing 30% FBS) was added to the lower chamber. After incubating at 37 °C for 24–36 h, migrated or invaded cells were fixed and stained with crystal violet for 15 min. Images were obtained from five random fields (× 100) in each well. Experiments were performed in triplicate.

### Cell viability assay

Cell viability was determined using the CCK-8 assay (Dojindo, Kumamoto, Japan). Transfected cells were seeded into 96-well plates (5 × 10^3^ cells/well) and incubated overnight. Then, CCK-8 solution was added to each well every 24 h. After incubation for additional 2 h at 37 °C, each sample was measured at 450 nm by a microplate reader (BioRad, Hercules, CA, USA).

### EdU assay

Cell proliferation was measured with an EdU assay kit (Ribo-Bio, Guangzhou, China). Experiments were performed according to the manufacturer’s instructions. The representative images were obtained using a Leica inverted fluorescence microscope.

### Colony-forming assay

After the indicated treatment, cells (1.5 × 10^2^/well) were added into six-well plates per well and cultured for 2 weeks. Cells were fixed with 100% methanol and stained with 5% crystal violet, and number of colonies per well were counted. Experiments were performed in triplicate.

### Reverse-transcription PCR

Total RNA was isolated from cells or human tissue using TRIzol Reagent (Takara, Tokyo, Japan) according to manufacturer’s instructions. cDNA was synthesized from total RNA (2 µg) using reverse transcription kit (Toyobo Life Science, Shanghai, China). qPCR was performed in triplicate using 1 µL of cDNA in a standard SYBR premix Ex Taq (Takara) on the CFX96 Real-Time PCR Detection System (Bio-Rad, Hercules, CA, USA). GAPDH served as an internal control. The following primers were used: GAPDH, 5′-AATGAAGGGGTCATTGATGG-3′, 5′-AAGGTGAAGGTCGGAGTCAA-3′;

ACTL6A, 5′-AGCCCTTAGGGTAGGAGTCG-3′, 5′-CAAGGGCTCCAACTTCATCT-3′; CTGF, 5′-AGGAGTGGGTGTGTGACGA-3′, 5′-CCAGGCAGTTGGCTCTAATC-3′; CYR61, 5′-GAGTGGGTCTGTGACGAGGAT-3′, 5′-GGTTGTATAGGATGCGAGGCT-3′; YAP, 5′-ACCCTCGTTTTGCCATGAAC-3′, 5′-TGTGCTGGGATTGATATTCCGTA-3′; and TAZ, 5′-GGCTGGGAGATGACCTTCAC-3′, 5′-CTGAGTGGGGTGGTTCTGCT-3′.

### Luciferase reporter assays

HEK293 cells were cotransfected with Firefly luciferase reporters and indicated plasmid using Lipofectamine 2000 (Invitrogen/Thermo Fisher Scientific), and luciferase assays were performed 24 h later using the Dual-Luciferase Reporter Assay Kit (Promega). *Renilla* activity was used to normalize luciferase reporter activity. For knockdown experiments, cells were transfected with indicated siRNA 24 h before plasmid transfection.

### Animal studies

To establish an intracerebral glioma model, control and modified glioma cell lines were implanted into 4-week-old female nude mice (*n* = 32; Shanghai SLAC Laboratory Animal Co., Ltd, Shanghai, China). Animals were divided into four groups (eight mice per group) and inoculated into the frontal lobe using a stereotactic apparatus (KDS310, KD Scientific, Holliston, MA, USA) with one of the following cell populations (1 × 10^6^ cells): U251-NC, U251-shACTL6A, U87MG-NC, and U87MG-ACTL6A. Animals displaying symptoms, such as severe hunchback posture, apathy, decreased motion, or activity, dragging legs, unkempt fur, or drastic loss of body weight were killed by cervical dislocation. Excised tumor tissues were further examined through hemtoxylin and eosin, and IHC staining

For subcutaneous glioma model, nude mice (*n* = 40) were divided into eight groups (U87MG-NC, U87MG-ACTL6A, U87MG-ACTL6A + shcontrol, U87MG-ACTL6A + shYAP/TAZ, U251-NC, U251-sh-ACTL6A, U251-sh-ACTL6A + control, and U251-sh-ACTL6A + YAP-5SA/TAZ-4SA, five mice per group). Cells were resuspended in PBS/Matrigel (BD Biosciences) at a density of 10^7^ cells/ml and injected into the right shoulder of the nude mice. Tumor tissues were excised and weighed 30 days after inoculation.

### Statistical analysis

Data were expressed as the mean ± SEM. The Student’s *t*-test for paired data was used to compare mean values. Analysis of variance was used to analyze the data of two groups with continuous variables. Survival curves were estimated by the Kaplan–Meier method and compared using the log-rank test. A two-tailed *χ*^2^-test was used to determine the association between ACTL6A and YAP/TAZ. Statistical analysis was conducted using GraphPad Prism version 7.00 software program for Windows (GraphPad, La Jolla, CA, USA). All tests were two-sided and *P*-values < 0.05 were considered to be statistically significant.

## Electronic supplementary material

SUPPLEMENTAL MATERIAL
