# Supplementary material for: Actin like-6A promotes glioma progression through stabilization of transcriptional regulators YAP/TAZ
Source: Cell Death Dis. 2018 May 3;9(5):517. doi: 10.1038/s41419-018-0548-3 (PMC5938705; doi:10.1038/s41419-018-0548-3)
Supplement: Supplementary file 1 — SUPPLEMENTAL MATERIAL [file 41419_2018_548_MOESM1_ESM.doc]

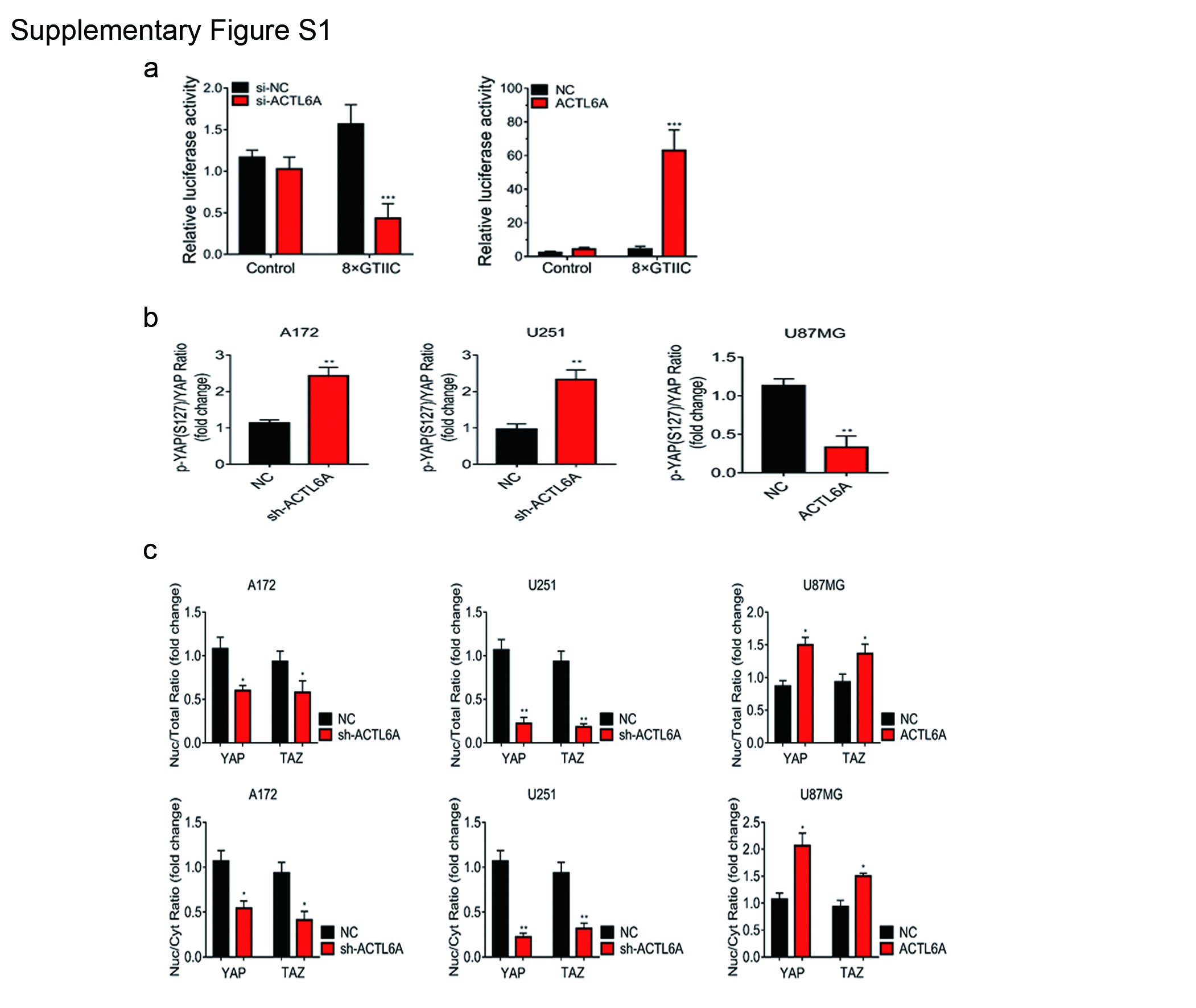


**Figure S1**

(a) Luciferase assay for 8xGTIIC-Lux or control reporter indicating YAP/TAZ dependent transcriptional activity by si-ACTL6A or ACTL6A treatment. Data are normalized to a Renilla reporter and to negative control (NC). (b) Image J was introduced to evaluate the western blot results in Figure 4c. Data are represented as the mean ± SEM. (c) Image J was introduced to evaluate the western blot results in Figure 4e. Graph showing the mean YAP and TAZ levels normalized to cytoplasmic (GAPDH) and nuclear (Histone-H3) markers and then to NC cells. Data are represented as the mean ± SEM. Student’s *t*-test: * *P* < 0.05, ** *P* < 0.01, *** *P* < 0.001.


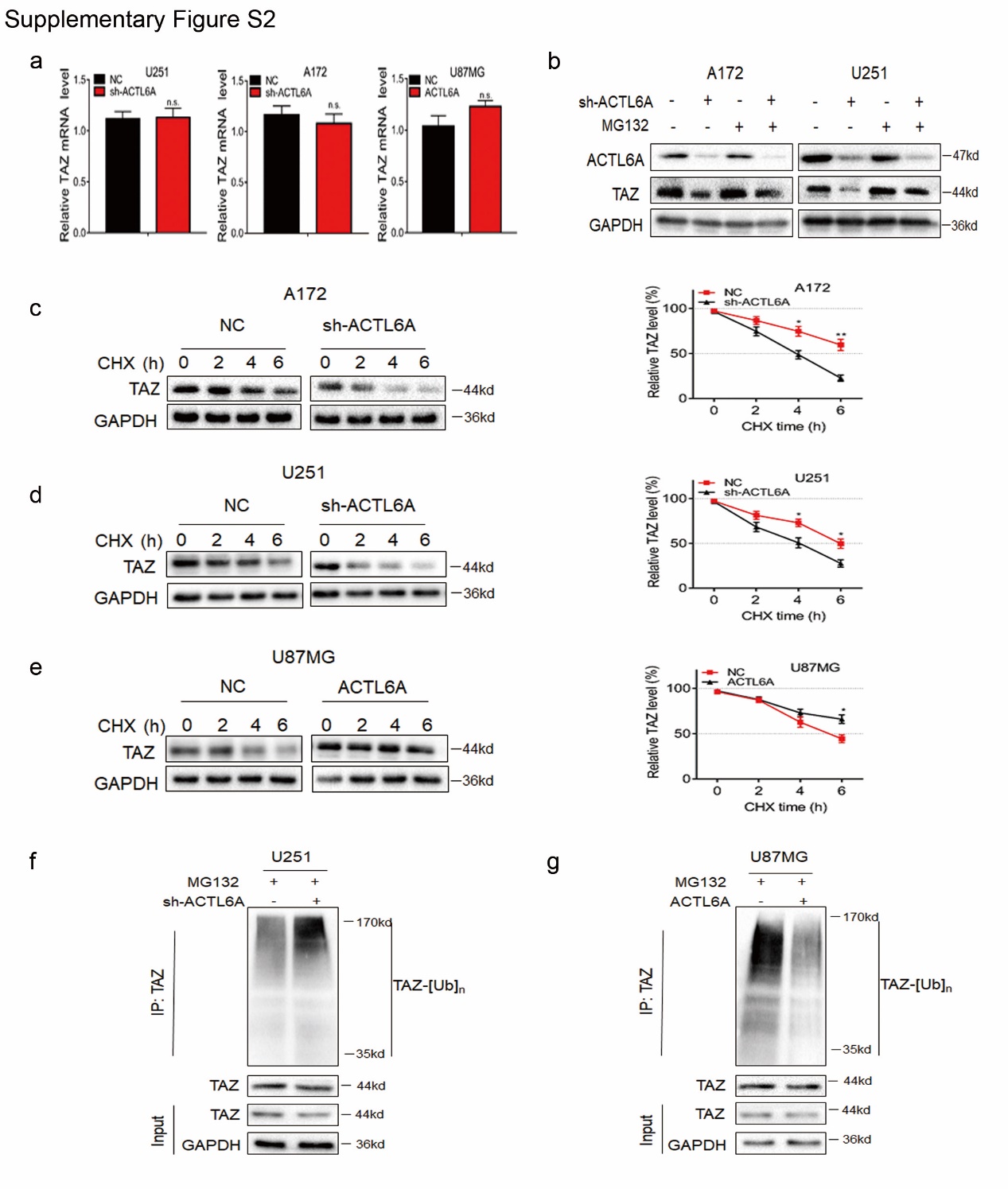


**Figure S2**

(a) qRT-PCR analysis of TAZ in A172- and U251-NC and -sh-ACTL6A and U87MG-NC and ACTL6A cells. Expression is normalized to GAPDH mRNA. Data are represented as the mean ± SEM. (b) Western blot analysis to evaluate TAZ levels in A172- and U251-NC and -sh-ACTL6A cells after MG132 (20 µM) treatment for 8 h. GAPDH was used as loading control. (c, d, e) Western blot analysis of TAZ protein in modified A172, U251 and U87MG cells treated CHX (25 µg/mL) for the indicated time. Line graph shows YAP levels normalized to GAPDH at the indicated time points (*n* = 4). Data are represented as the mean ± SEM. (f, g) Western blot analysis of TAZ IPs performed on lysates prepared from U251-NC and -sh-ACTL6A and U87MG-NC and -ACTL6A cells treated with MG132 (20 µM) for 8 h to examine endogenous TAZ ubiquitination. Student’s *t*-test: n.s. = not significant, **P* < 0.05, ** *P* < 0.01.


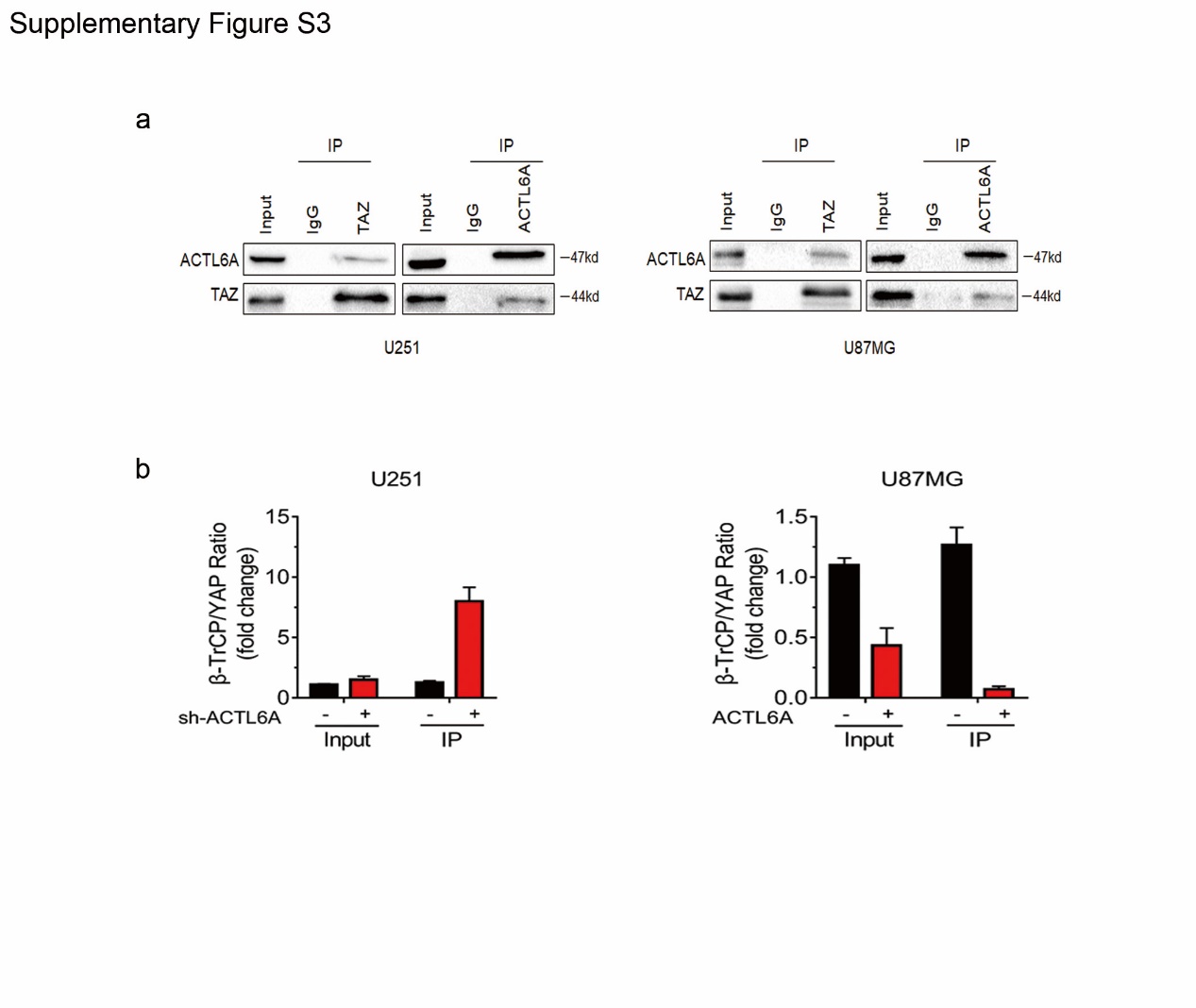


**Figure S3**

(a) Western blot analysis of co-precipitating proteins in IPs performed using anti-TAZ or -ACTL6A antibody on lysates prepared from U251 and U87MG cells. (b) Image J was introduced to evaluate the western blot results in Figure 6e and 6f. Graph showing the mean β-TrCP normalized to YAP and then to NC cells. Data are represented as the mean ± SEM.


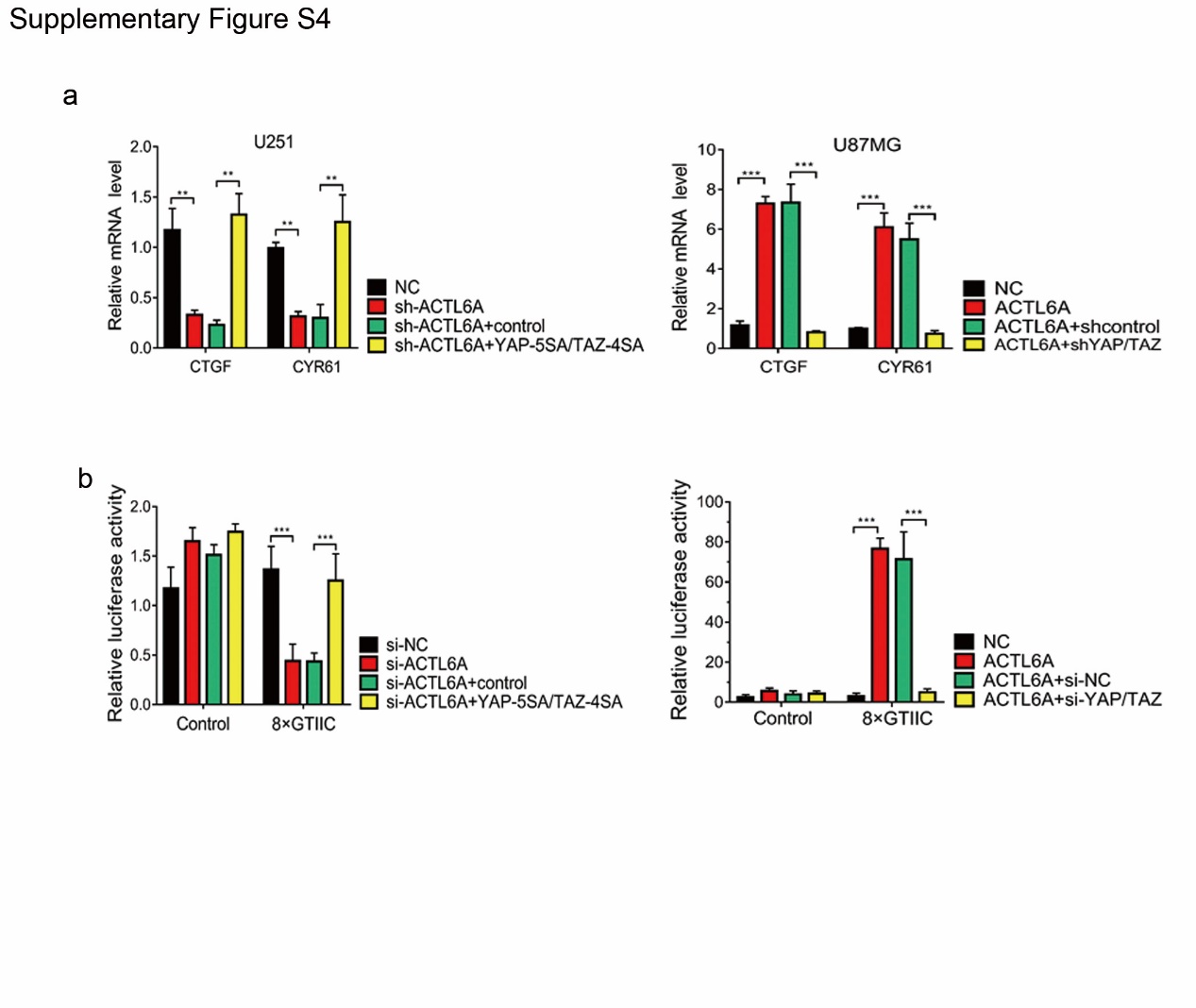


**Figure S4**

(a) qRT-PCR analysis of *CTGF* and *CYR61* in ACTL6A-interfered glioma cells with YAP/TAZ knockdown or active forms ectopic expression. Expression is normalized to GAPDH mRNA. Data are represented as the mean ± SEM. (b) Luciferase assay for 8xGTIIC-Lux or control reporter indicating YAP/TAZ dependent transcriptional activity by indicated treatment. Data are normalized to a Renilla reporter and to negative control (NC). Student’s *t*-test: *** *P* < 0.001.


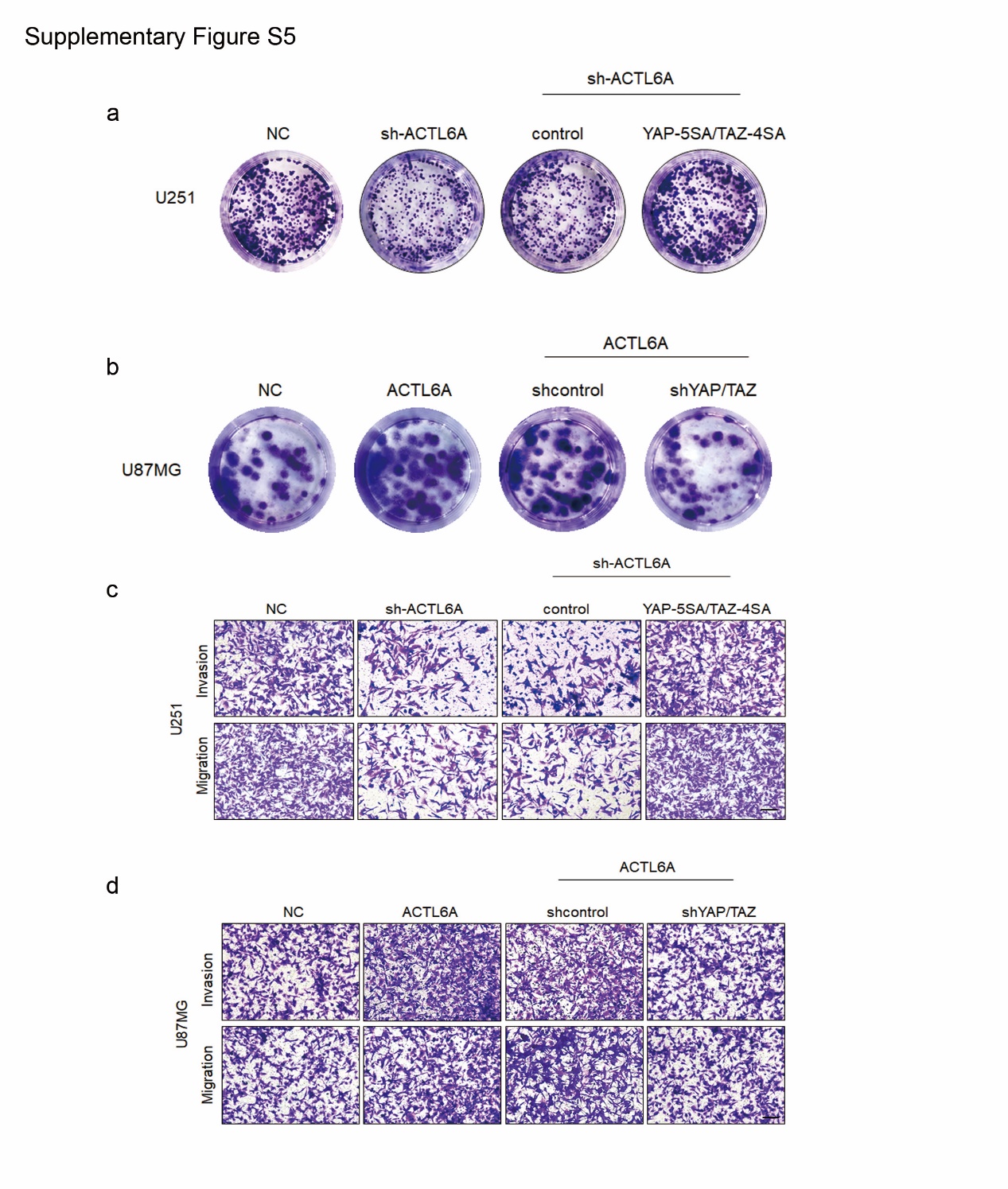


**Figure S5**

(a, b) Representative images of colony forming assay performed on ACTL6A-interfered glioma cells with YAP/TAZ knockdown or active forms ectopic expression.

(c, d) Representative images of transwell migration and invasion assays performed in ACTL6A-interfered glioma cells with YAP/TAZ knockdown or active forms ectopic expression.

**
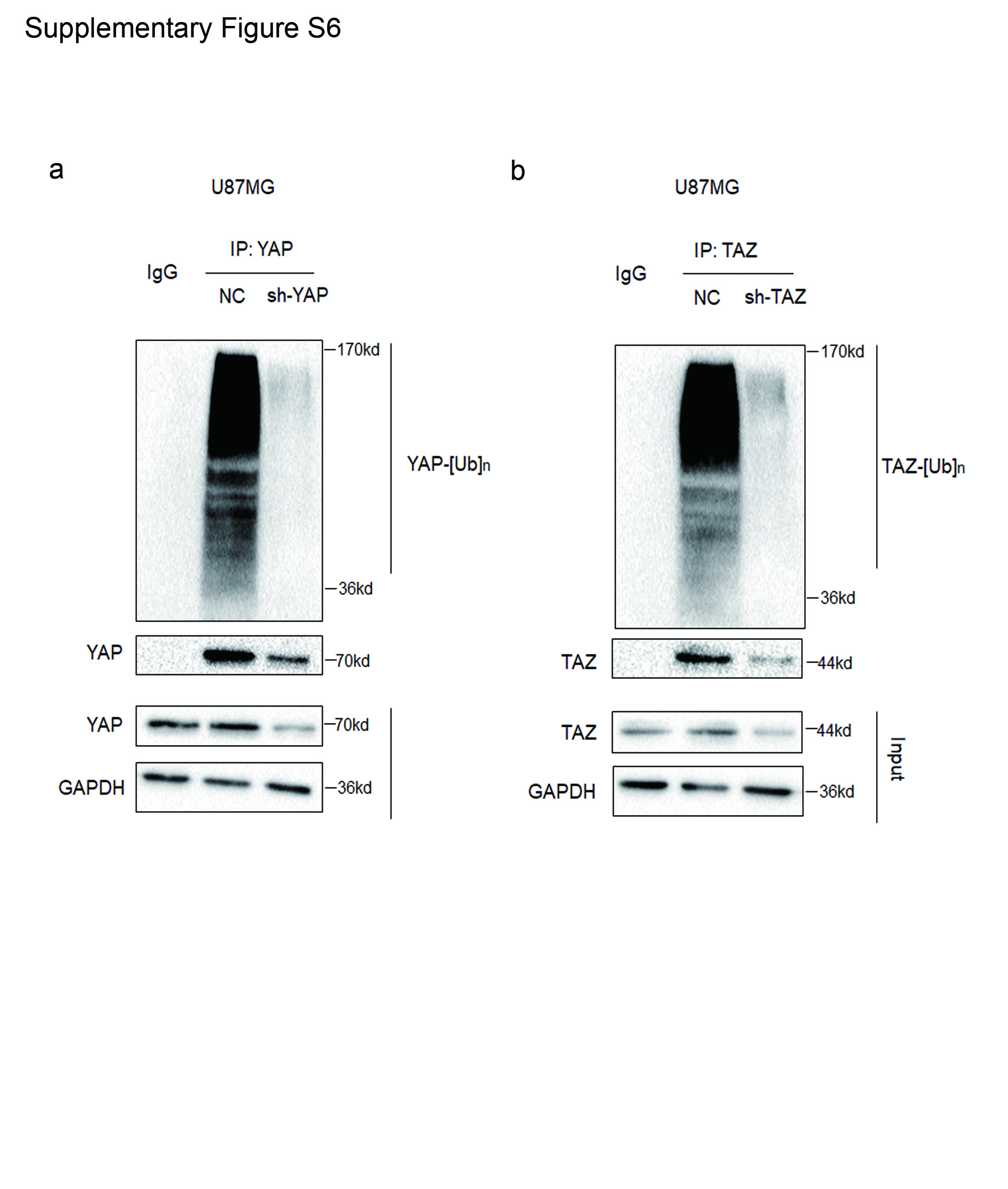
**

**Figure S6**

(a, b) Western blot analysis of YAP/TAZ IPs performed on lysates prepared from U87MG-NC and sh-YAP/TAZ cells treated with MG132 (20 µM) for 8 h to examine endogenous YAP/TAZ ubiquitination. Normal rabbit IgG groups were served as negative control.

**
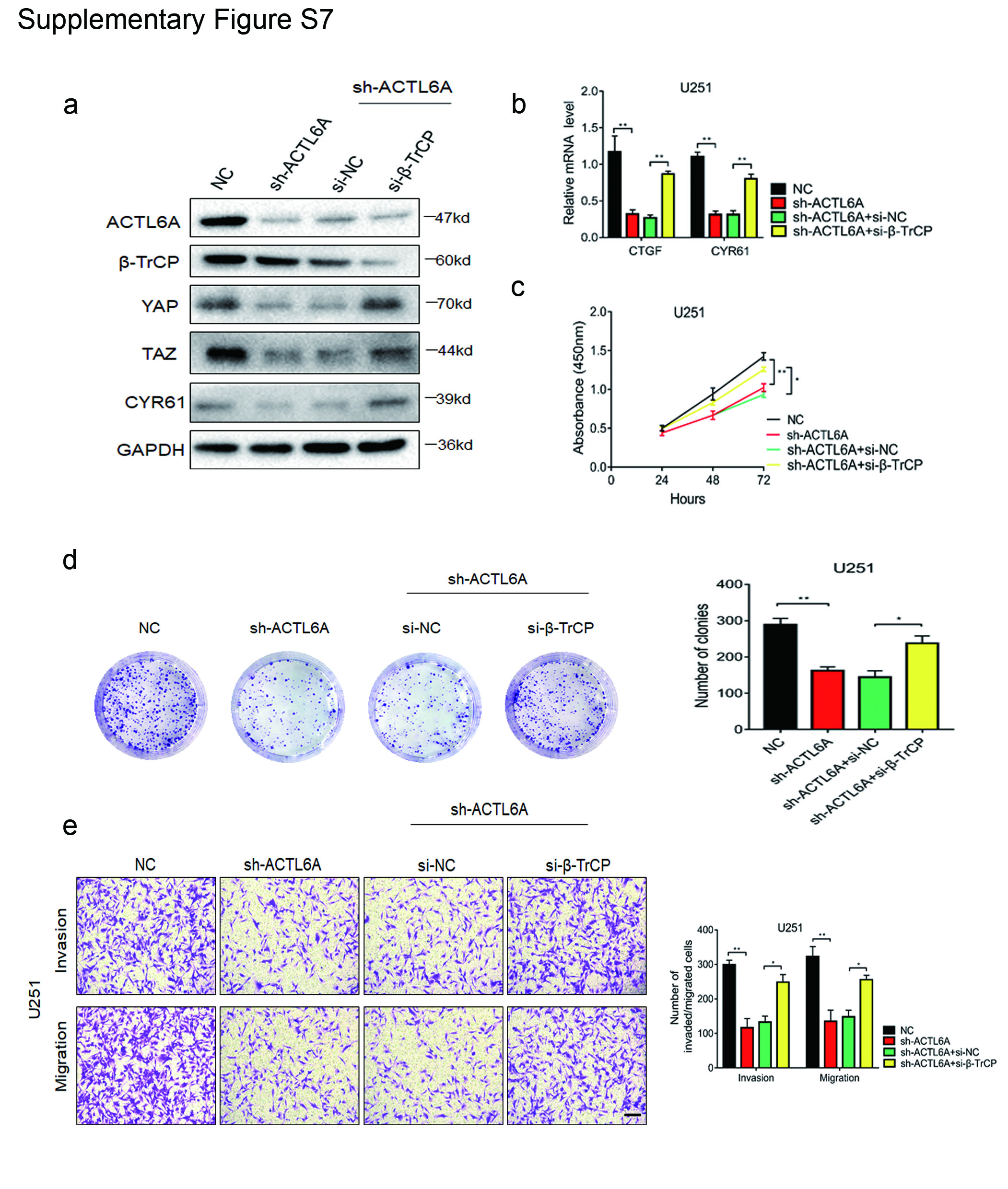
**

**Figure S7**

(a) Western blot analysis of lysates prepared from ACTL6A-interfered glioma cells with β-TrCP knockdown. GAPDH was used as loading control. (b) qRT-PCR analysis of *CTGF* and *CYR61* in ACTL6A-interfered glioma cells with β-TrCP knockdown. Expression is normalized to GAPDH mRNA. Data are represented as the mean ± SEM. (c) CCK8 assay performed on ACTL6A-interfered glioma cells with β-TrCP knockdown. Data are represented as the mean ± SEM. (d) Colony forming assay performed on ACTL6A-interfered glioma cells with β-TrCP knockdown. Graphic representation of the colony numbers under each condition. Data are represented as the mean ± SEM. (e) Representative images of transwell migration and invasion assays performed in ACTL6A-interfered glioma cells with β-TrCP knockdown. Graphic representation of migrated and invaded cells counts from transwell assays performed in ACTL6A-interfered glioma cells with β-TrCP knockdown. Data are represented as the mean ± SEM from three independent experiments. Scale bars, 100 µm. Student’s *t*-test: **P* < 0.05, ** *P* < 0.01.
